# Mycophenolate mofetil induces angiogenesis disorders in zebrafish embryos by inhibiting the mitogen-activated protein kinase signaling pathway

**DOI:** 10.1186/s40659-026-00717-7

**Published:** 2026-07-17

**Authors:** Mengqi Wan, Zhonghao Xiao, Xiaowen Shi, Zhijun Ye, Jiejun Liu, Xuyang Liu, Huimin Li, Xinjun Liao, Shouhua Zhang, Juhua Xiao, Zigang Cao

**Affiliations:** 1 Jiangxi Engineering Laboratory of Zebrafish Modeling and Drug Screening for Human Diseases, Jiangxi Key Laboratory of Developmental Biology of Organs and Epigenetics, Key Laboratory of Jiangxi Province for Biological Invasion and Biosecurity, College of Life Sciences, Clinical Research Center of Jinggangshan University, Jinggangshan University, Ji’an, 343009 China; 2 Key Laboratory of Developmental Biology of Organs and Epigenetics, Clinical Research Center of Jinggangshan University, Jinggangshan University, Ji’an, 343009 China; 3 Department of General Surgery, Jiangxi Provincial Children’s Hospital, The Affiliated Children’s Hospital of Nanchang Medical College, Nanchang, Jiangxi China; 4 Department of Ultrasound, Jiangxi Provincial Maternal and Child Health Hospital, Nanchang, 330006 Jiangxi China; 5 Jiangxi Engineering Laboratory of Zebrafish Modeling and Drug Screening for Human Diseases, Jiangxi Key Laboratory of Developmental Biology of Organs and Epigenetics, College of Life Sciences, Clinical Research Center of Jinggangshan University, Jinggangshan University, Ji’an, 343009 China

**Keywords:** Mycophenolate mofetil, Vascular development, MAPK signaling, Zebrafish

## Abstract

As a commonly used immunosuppressant, mycophenolate mofetil (MMF) is widely prescribed after organ transplantation because it is not toxic to the liver and kidney. However, its use is associated with certain gastrointestinal toxicity and increased risk of opportunistic infections. Meanwhile, whether it affects vascular development is unclear. Herein, a zebrafish model was utilized to evaluate whether MMF affects vascular development, and the results showed that MMF exhibited teratogenicity, shortened the body length, caused pericardial edema, and delayed yolk sac absorption in zebrafish. In addition, although Intersegmental vessels showed hyperbranching, opposite changes were observed in cerebrovascular, with cerebrovascular inhibition. Moreover, MMF exposure upregulated oxidative stress levels, inhibited notch signaling and mitogen-activated protein kinase (MAPK) signaling, and affected the expression of vascular endothelial growth factor and receptor tyrosine kinase. Treatment with the antioxidant astaxanthin and the notch signaling pathway activator sodium valproate did not rescue the vascular development defects, indicating that MMF affects multiple signal pathways. However, the treatment of MMF-exposed zebrafish with guanosine and the MAPK signaling activators diprovocim significantly rescued the angiogenesis-related defects, suggesting that MMF induces ISV hyperbranching in zebrafish by inhibiting MAPK signaling and inosine 5′-monophosphate dehydrogenase (IMPDH).

## Introduction

Mycophenolate mofetil (MMF), a relatively new systemic immunosuppressant, is a prodrug of mycophenolic acid, which was initially widely used for treating various skin diseases. In 1995, MMF was approved by the US Food and Drug Administration for the prevention of kidney transplant rejection and is currently used in immunosuppressive regimens for various organ transplants. MMF inhibits the synthesis of inosine 5′-monophosphate dehydrogenase (IMPDH), a key enzyme in the de novo synthesis pathway of guanine nucleotide, in a noncompetitive and reversible manner, thereby inhibiting the proliferation of B and T lymphocytes [33, 44]. Compared with other immunosuppressants, MMF does not cause liver and kidney toxicity and exhibits lower bone marrow toxicity and gastrointestinal toxicity such as diarrhea, gastritis, and others. In addition, MMF can increase the risk of cytomegalovirus infection, leukopenia, and mild anemia [32, 35]. Of note, the occurrence of toxic effects may be correlated with age to some extent; the probability of toxicity is higher in younger patients. Therefore, MMF is used more cautiously in young patients. Unfortunately, few studies have assessed the vasotoxic effects of MMF.

Zebrafish, a powerful and recognized animal research model for toxicology, is widely used in biomedical research and drug screening. Unlike other animal models, zebrafish is small in size, economical, and easy to raise. In addition, in zebrafish, the embryo is transparent, in vitro fertilization is feasible, and the reproductive ability is strong. Indeed, a pair of adult fish can produce 200–300 embryos a day, thereby reducing the cost of drug research and screening and shortening the drug experiment cycle [16, 28]. Zebrafish embryos develop outside the body, and their development can be easily monitored from the earliest stages. This is different from mammalian embryos, which develop in the womb. Zebrafish can survive even with developmental malformations, organ loss, or organ dysfunction [15], and its genes have a high degree of homology with human genes [24]. Finally, zebrafish have a closed circulatory system, and the anatomy, and molecular mechanisms of its vascular development are very similar to those of humans and other higher vertebrates [13, 29]. Thus, zebrafish has been widely used in the assessment of cardiovascular development and screening of drugs for cardiovascular diseases.

In the present study, zebrafish was used as the model animal for studying vascular toxicity, and the results revealed that different degrees of malformations occurred during the development of zebrafish arterial intersegmental and cerebrovascular at different drug concentrations. In addition, MMF exposure led to expression disorders of genes associated with vascular development, downregulation of IMPDH expression, and upregulation of oxidative stress level. Meanwhile, the study results revealed that guanosine and MAPK activation effectively rescued MMF-induced vascular development defects in zebrafish. These findings suggest that MMF induces defects in the vascular development of zebrafish by inhibiting IMPDH and MAPK signaling pathway.

## Materials and methods

### Chemicals

For this study, MMF was purchased from Shanghai Yuanye Biotechnology Co., Ltd. (CAS NO.115007-34-6; >98% Assay), and the working solution was prepared with dimethyl sulfoxide(DMSO) at the final concentrations of 10, 20, and 40 µg/L, the control group was treated with DMSO alone, at the same volume as that added in the highest MMF exposure group. TRIzol kits were purchased from Takara (Dalian, China), reverse transcription kits and qPCR kits were bought from Quanshijin (Beijing, China), and guanosine was obtained from Solaibao (Beijing, China).

### Animals

Wild-type AB zebrafish *Tg(TP1:EGFP)* and *Tg(fli1a: EGFP)* used in this study were purchased from the China Zebrafish Resource Center; the lines are cataloged in the ZFIN database as Zf3185Tg for *Tg(TP1:EGFP)* and y1Tg for *Tg(fli1a: EGFP)* [17, 31], and raised in a zebrafish recirculating culture system at a standard temperature of 28 °C and light-to-dark ratio of 14 h:10 h.They were placed in the mating tank (male: female ratio of 1:1) overnight, and the embryos were collected 30 min after spawning the next day. The collected embryos were placed in a petri dish containing an embryo culture medium, and the petri dish was placed in an embryo incubator for 24 h.

### Exposure to MMF

Zebrafish at 24 hpf were collected from the embryo incubator, and healthy embryos at the same developmental stage were selected under an ordinary light microscope. The selected embryos were placed in a 6-well plate, with 20 seeds per well. The final working concentrations of the drug were determined based on the outcomes of preliminary assays assessing survival rates and the severity of phenotypic defects. The control, 10 µg/L, 20 µg/L, and 40 µg/L groups were set up, and 5 mL of 0.003% PTU culture medium (Sigma, USA) was added to each well for inhibiting pigment growth. PTU and MMF were changed daily for 3 consecutive days of drug exposure. After 3 days, the development of arterial and cerebrovascular as well as the gene expression of vascular development and related signaling pathways in zebrafish larvae were observed and recorded. Each group of experiments was repeated at least three times.

### Effect of MMF exposure on vascular development

The Tg(*fli1a: EGFP*) zebrafish embryos were treated with MMF, and the developmental morphology of zebrafish embryos, intersegmental blood vessels, and cerebrovascular under white light and fluorescence were photographed and recorded using a fluorescence microscope and confocal microscope, respectively. In addition, the heart rate (20 s/time), body length (µm), yolk sac area (µm^2^), pericardial area (µm^2^) of zebrafish embryos were calculated using the Zeiss 20 system, and statistical plots were drawn. All experiments were repeated three times.

### mRNA expression

Total RNA extracted from zebrafish larvae in each group was collected and reverse-transcribed to obtain cDNA, which was subjected to real-time PCR for the following genes: vascular endothelial growth factorVEGFaa, VEGFab, VEGFc, and VEGFd; the tyrosine kinase receptor genes Flt1, Flt4, Kdr, and Kdrl; and the notch signaling pathway-related genes notch 1a and Her1. The expression levels of Her6, MAPK signal-related genes (MAPK1, MAPK3, MAPK8b, MAPK9, p38a, and p38b), and IMPDH were plotted using GraphPad Prism 7.

### Oxidative stress level check

Wild-type AB zebrafish embryos in each group were collected after 72 h of drug treatment, washed three times in PBS, stained with ROS, and analyzed using Zeiss microscope (Discovery. V20). At the same time, the total protein of zebrafish larvae treated with the drug for 72 h was collected, and the activity of oxidative stress-related enzymes such as superoxide dismutase (SOD) and malondialdehyde (MDA) was determined using absorbance via SpectraMax^®^ iD3 Multi Mode. To verify whether zebrafish vascular toxicity caused by MMF exposure is mediated by oxidative stress signaling pathway, astaxanthin was used to rescue the MMF-exposed zebrafish larvae, and laser confocal microscopy was used to assess the larval blood vessels of each group. All experiments in each group were repeated three times.

### Rescue experiments

The Tg(*TP1:EGFP*) and Tg(*fli1a: EGFP*) zebrafish embryos were treated with MMF and photographed and assessed after 72 h of drug treatment. The MMF-exposed zebrafish larvae were treated with sodium valproate, an activator of the notch signaling pathway, and vascular changes in zebrafish larvae were observed and recorded via laser confocal microscopy after 72 h of drug treatment. The results of each experimental group were subjected to fluorescence quantitative analysis.

The Tg(*fli1a: EGFP*) zebrafish embryos were simultaneously treated with 40 µg/L MMF and guanosine at 72 hpf and analyzed using Leica M205FA. The controls were 40 µg/L MMF treatment, PTU only treatment, and guanosine only treatment groups.

The Tg(*fli1a: EGFP*) embryos were simultaneously treated with 40 µg/L MMF and the MAPK signaling pathway activator diprovocim (Dip) at 72 hpf and analyzed using Leica M205FA. The controls were 40 µg/L MMF treatment, PTU only treatment, and Dip only treatment groups.

### Statistical analysis

The control and different experimental groups were statistically analyzed using analysis of variance and t-test. All data are expressed as the mean ± standard deviation, and p-value < 0.05, < 0.01, and < 0.001 indicated statistical significance.

## Results

### MMF exposure induces vascular development defects in zebrafish

**Fig. 1:**
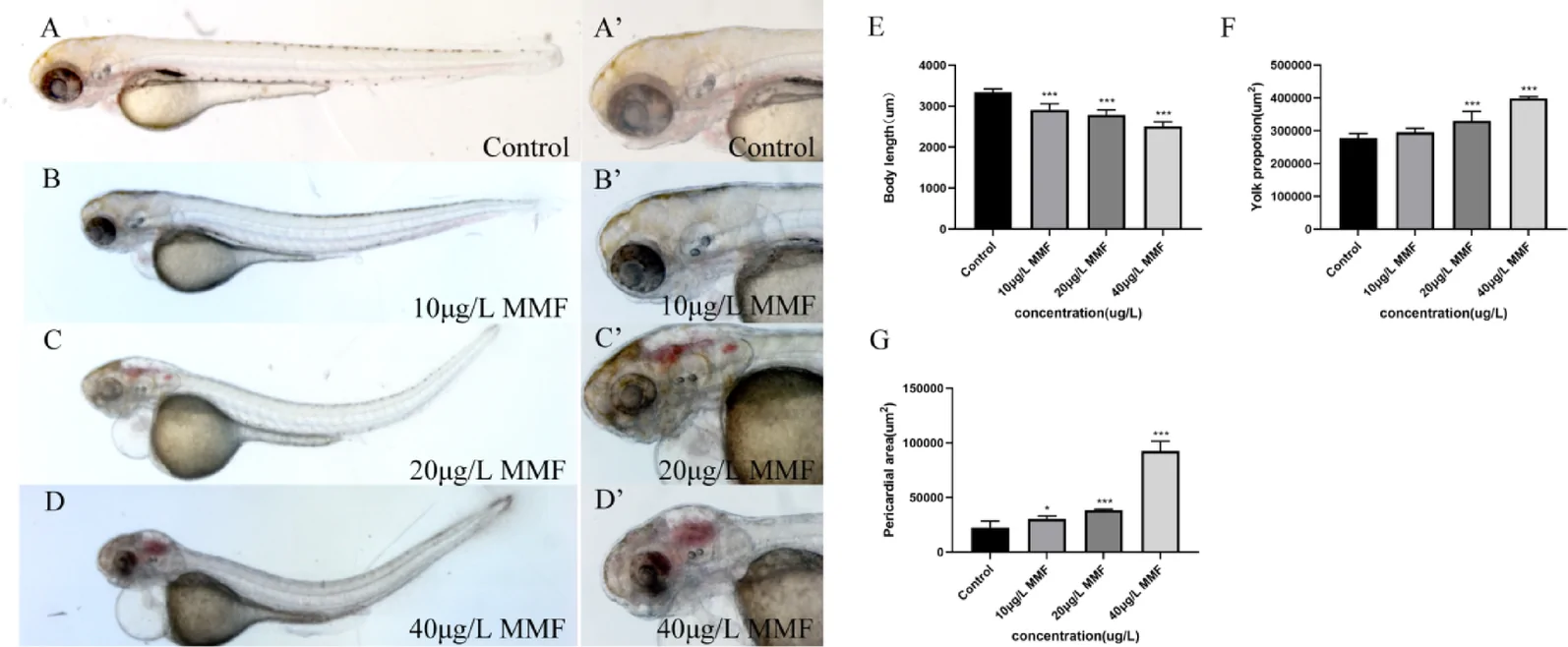
Effects of MMF on embryonic development in zebrafish. **A**-**D**^,^ Morphological changes such as shortened body length, cerebral hemorrhage, and pericardial edema in zebrafish exposed to 10, 20, and 40 µg/L MMF for 72 h. **E**-**G** Body length, yolk sac area, and pericardium area of zebrafish larvae at 72 h of MMF exposure. The data represents the mean ± S.D. *p < 0.05, **p < 0.01, ***p < 0.001

To investigate whether MMF exposure affect vascular development, 72 hpf zebrafish embryos were treated with MMF, and the results revealed that compared with the control group, the MMF-treated zebrafish larvae had significantly shorter body length, cerebral hemorrhage, pericardial edema, and delayed yolk sac absorption, particularly in the 10 µg/L MMF group (Fig. 1A-D’). With the increase in drug concentration, the body length decreased (Fig. 1E), yolk sac absorption was delayed (Fig. 1F), and pericardial area increased (Fig. 1G).

**Fig. 2:**
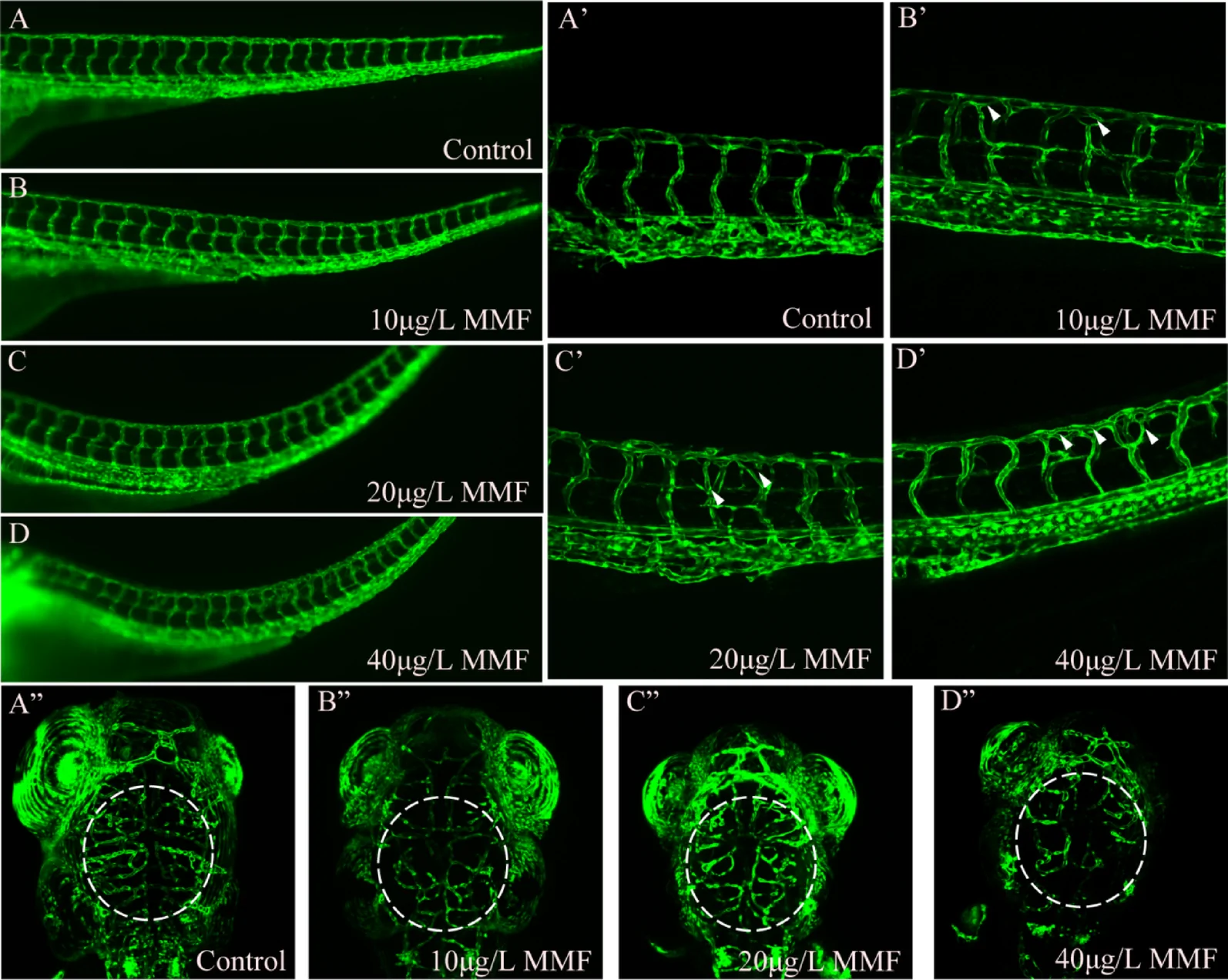
MMF exposure affect vascular development. **A**-**D**^,^ Intersegmental vessels of zebrafish larvae after 72 h MMF exposure. **A**^, ,^ -**D**^, ,^ cerebrovascular of zebrafish larvae after 72 h MMF exposure

Under laser confocal microscopy, zebrafish intersegmental blood vessels showed irregular hyperbranching (Fig. 2A-D) and the appearance of small branch vessels increased with the increase in drug concentration with white arrows indicating abnormal hyperbranching vessels in (Fig. 2A’-D’). By contrast, an opposite trend was observed in the cerebrovascular vessels; the trend of inhibition of cerebrovascular was initiated from the 10 µg/L group. In addition, the cerebrovascular development was irregular, in sharp contrast to the symmetry of the cerebrovascular development in the control group, with white circles marking the impaired cerebrovascular structures in (Fig. 2A’’-D’’).

### Effect of MMF exposure on oxidative stress levels

**Fig. 3:**
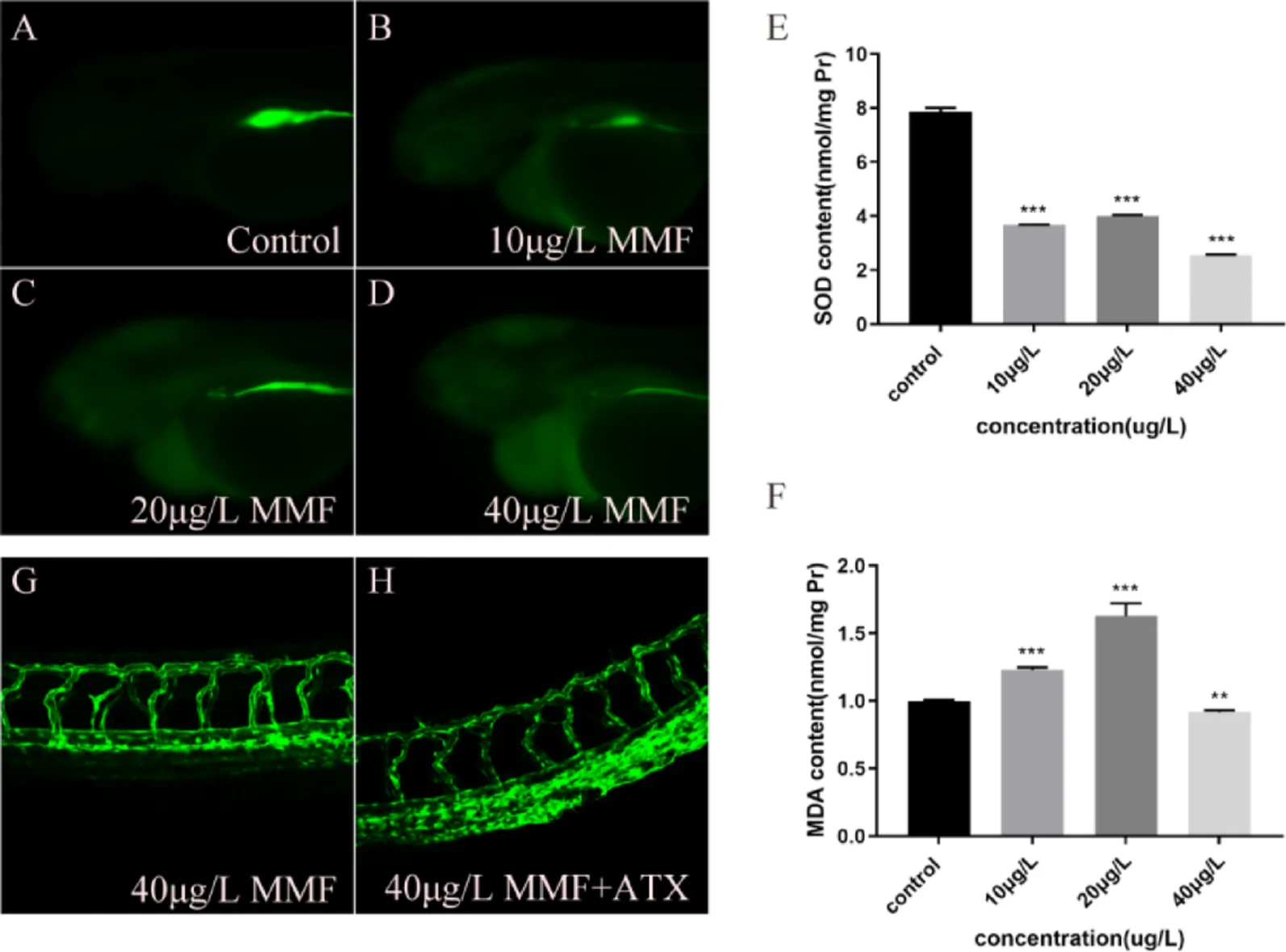
Effect of MMF exposure on oxidative stress levels. **A**-**D** ROS production level evaluated via DCFH-DA staining after 72 h MMF exposure. **G**-**H** ATX was used to rescue Tg(*fli1a: EGFP*) zebrafish after 72 h MMF exposure. [E] SOD activity after 72 h MMF exposure. **F** MDA activity after 72 h MMF exposure. The data represents the mean ± S.D. *p < 0.05, **p < 0.01, ***p < 0.001

Oxidative stress plays an important role in the physiological and pathological mechanisms of all aerobic organisms [29]. The ROS staining of MMF-exposed wild-type AB zebrafish showed that with the increase in drug concentration, green fluorescence increased (Fig. 3A-D), indicating that oxidative stress was upregulated. The assessment of the oxidative stress-related enzyme activity indices Superoxide Dismutase(SOD) and Malondialdehyde(MDA) revealed that SOD showed a downward trend (Fig. 3E) and MDA exhibited an upward trend to varying degrees (Fig. 3F). The antioxidant astaxanthin had no effect on developmental defects, and the articular vessels of zebrafish larvae treated with astaxanthin still showed irregular hyperplasia (Fig. 3G, H), which indicated that MMF-induced angiodevelopmental toxicity in zebrafish was caused by the upregulation of oxidative stress and was not associated with angiogenesis.

### Effect of MMF exposure on the notch signaling pathway

**Fig. 4:**
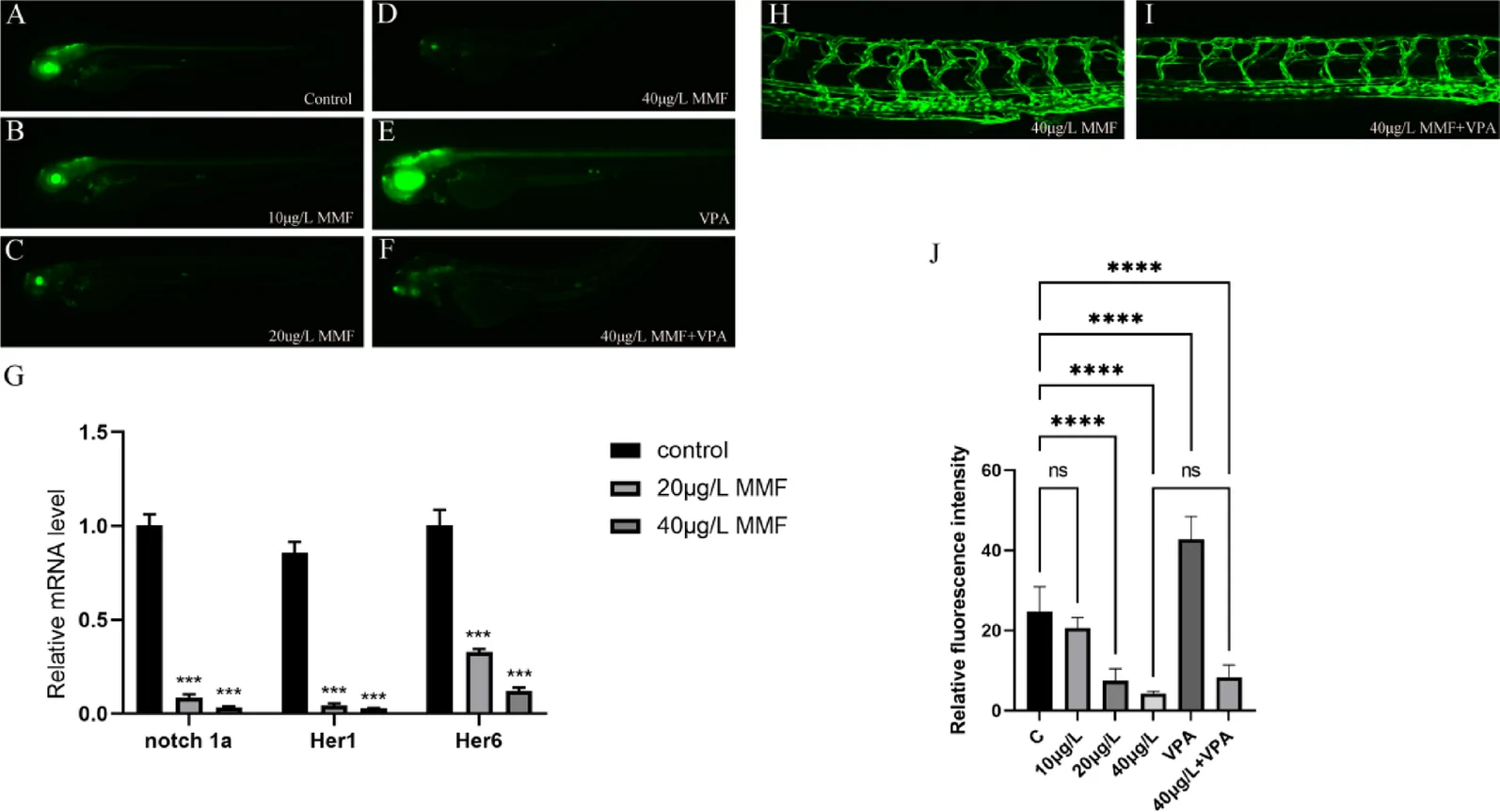
Effect of MMF exposure on the notch signaling pathway. **A**-**D** Representative maps in Tg(*TP1:EGFP*) zebrafish after 72 h exposure to different concentrations of MMF. **E**-**F** VPA was used to rescue Tg(*TP1:EGFP*) zebrafish after 72 h MMF exposure. **G** Changes in expression of genes involved in the notch signaling pathway after after 72 h MMF exposure. **H**-**I** VPA was used to rescue Tg(*fli1a: EGFP*) zebrafish after 72 h MMF exposure. The data represents the mean ± S.D. *p < 0.05, **p < 0.01, ***p < 0.001

To assess the mechanism of MMF-induced vascular development defects in zebrafish, the Tg(*TP1:EGFP*) zebrafish line was used to evaluate the level of Notch signaling. The results revealed that zebrafish treated with MMF exhibited reduced fluorescence (Fig. 4A-D), and with the increase in concentration, the fluorescence of the 20 and 40 µg/L groups almost disappeared.Meanwhile, the fluorescence intensity of the Tg(*TP1:EGFP*) zebrafish larvae in MMF + sodium valproate group was slightly enhanced by the notch signaling pathway activator sodium valproate (Fig. 4E, F); however, the vascular development of Tg(*fli1a: EGFP*) zebrafish was not rescued. The articular vessels of zebrafish larvae treated with MMF showed varying degrees of hyperplasia (Fig. 4H, I), We performed fluorescence quantification on zebrafish from all experimental groups, and the results showed that MMF exposure inhibited Notch signaling in a dose-dependent manner. However, the use of the Notch signaling activator sodium valproate could not reverse this phenomenon(Fig. 4J). The expression of the notch signaling pathway-related genes notch 1a, HER1, and HER6 was decreased to varying degrees (Fig. 4G). indicating that MMF exposure inhibited notch signaling but larval angiogenesis was not associated with notch signaling.

### Analysis of transcriptional levels of the effect of MMF exposure on Vegf and Mapk signaling

**Fig. 5:**
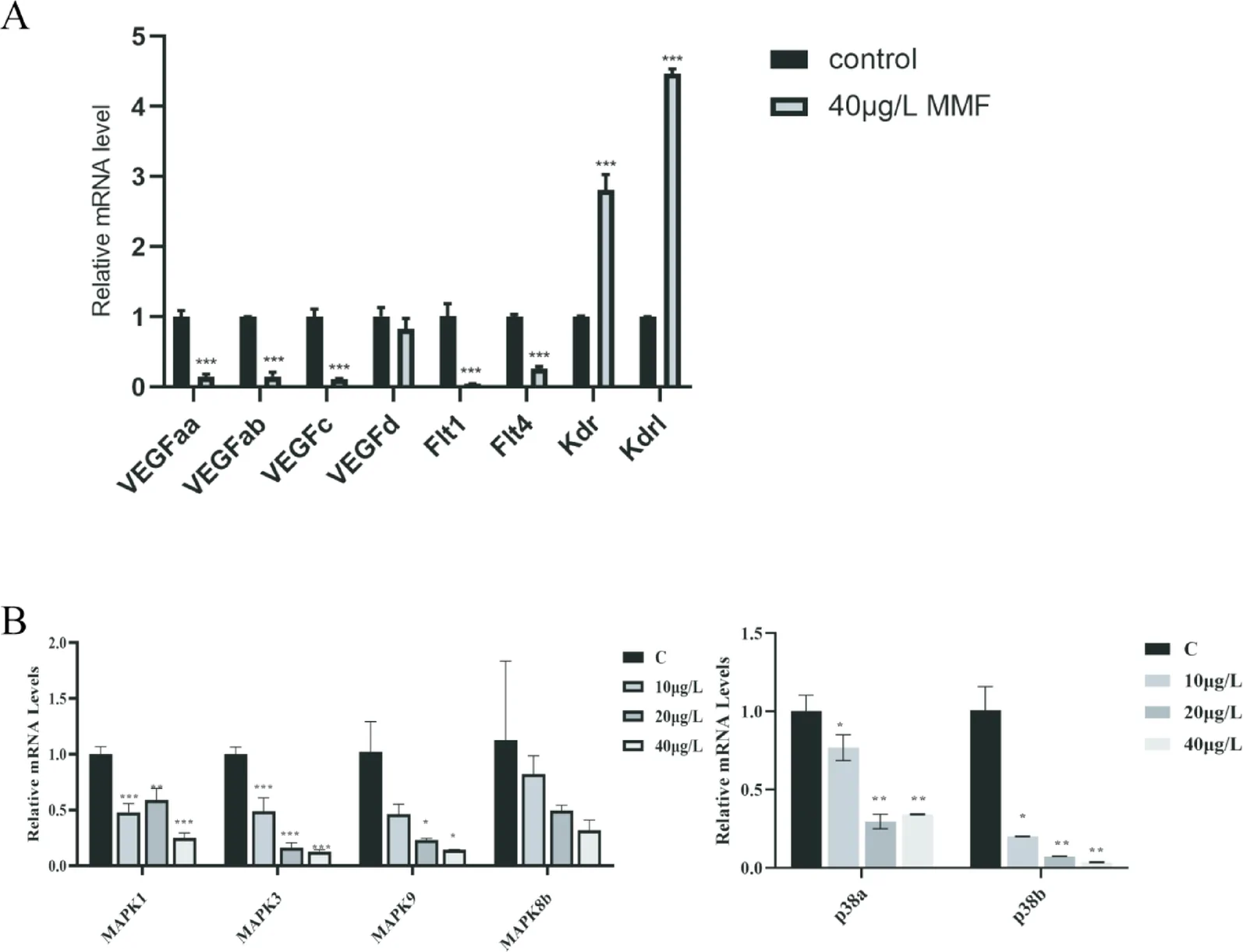
Analysis of transcriptional levels of the effect of MMF exposure on VEGF and MAPK after 72 h MMF exposure. **A** Changes in VEGF signaling transcript levels. **B** Changes in MAPK signaling pathway transcript levels. The data represents the mean ± S.D. *p < 0.05, **p < 0.01, ***p < 0.001

To investigate whether MMF exposure-induced defects in zebrafish vascular development are related to Vegf and Mapk signaling, qPCR experiments were performed to evaluate VEGF-related genes (VEGFaa, VEGFab, VEGFc, and VEGFd), related receptor tyrosine kinase-related genes (Flt1, Flt2, Kdr, and Kdrl), and MAPK signaling-related genes (MAPK1, MAPK3, MAPK8b, MAPK9, p38a, and p38b). The results showed that the expression of VEGF and related receptor tyrosine kinases was affected (Fig. 5A) and that the MAPK signaling-related genes were downregulated to varying degrees (Fig. 5B).

### Guanosine effectively rescued the MMF-induced excessive vascular branching

**Fig. 6:**
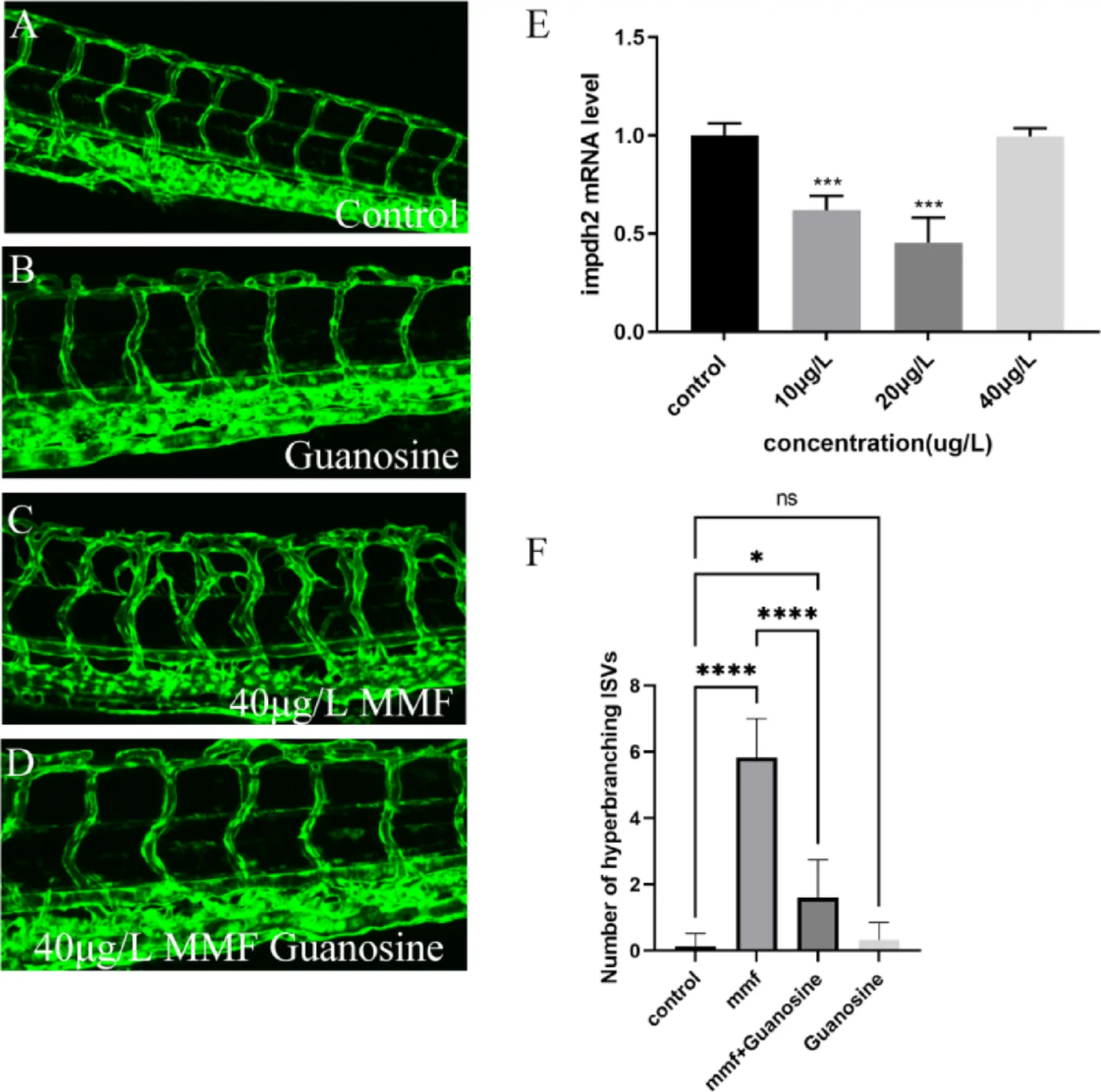
Guanosine effectively rescue the MMF-induced vascular development defects. **A**-**D** Guanosine was used to rescue Tg(*fli1a: EGFP*) zebrafish after 72 h MMF exposure. **E** Changes in expression of impdh2 in zebrafish after 72 h exposure to different concentrations of MMF. The data represents the mean ± S.D. *p < 0.05, **p < 0.01, ***p < 0.001.

To investigate the mechanism by which MMF induces vascular developmental defects in zebrafish, the expression level of IMPDH2 in zebrafish was assessed. The results showed that MMF significatily downregulated the expression level of IMPDH2 (Fig. 6E). In the MMF-treated group, obvious vascular hyperbranching and disorganization were observed (Fig. 6A, B,F). In contrast, guanosine effectively rescued ISV hyperbranching induced by MMF (Fig. 6B-D, F). These findings indicate that MMF inhibits the expression of IMPDH2, and guanosine alleviates the vascular developmental defects induced by MMF in zebrafish.

### Activation of MAPK signaling pathway rescued the vascular development defects induced by MMF

**Fig. 7:**
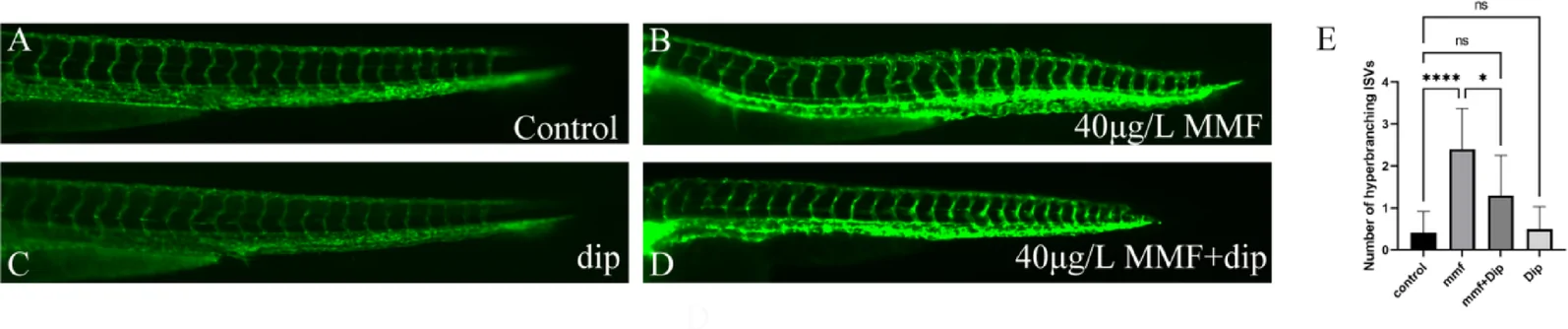
Activation of MAPK signaling pathway rescued the vascular development defects induced by MMF. **A**-**D** Tg(*fli1a: EGFP*) zebrafish were co-treated with DIP (diprovocim) and MMF for 72 h. **E** Analysis of intersegmental vascular changes in zebrafish after 72 h co-treatment with DIP (diprovocim) and MMF. The data represents the mean ± S.D. *p < 0.05, **p < 0.01, ***p < 0.001

Due to the downregulation of The MAPK signaling pathway by MMF, we used diprovocim, a MAPK signaling pathway activator, to treat MMF-induced vascular developmental defects in zebrafish (Fig. 7A-D). The results showed that diprovocim effectively rescued vascular disturbances compared with the MMF group (Fig. 7E), suggesting that MMF-induced angiogenesis defects are associated with MAPK signaling.

## Discussion

Mycophenolate (MMF) is a commonly used immunosuppressant that blocks purine biosynthesis. thereby inhibiting the proliferation of T and B lymphocytes is different from other commonly used immunosuppressants, such as calcineurin inhibitors. By contrast, MMF is not nephrotoxic, indicating a greater safety profile than calcineurin inhibitors after organ transplantation. Thus, MMF is often used to prevent allogeneic transplant rejection after liver, kidney, or heart transplantation. Meanwhile, MMF can also play a significant role in the treatment of autoimmune diseases such as lupus and myasthenia gravis [30, 37]. However, the side effects of MMF should be considered, as it may cause gastrointestinal side effects and increase the risk of opportunistic infections [9, 42]. However, the potential vasotoxicity of MMF during embryonic development remains poorly characterized. Using zebrafish as a model, we found that MMF exposure caused excessive vascular branching and increased venous sprouting, yet reduced cerebrovascular growth, accompanied by downregulation of multiple vascular development-related genes, as well as significant inhibition of the Notch, IMPDH, and MAPK signaling pathways. Rescue experiments using sodium valproate (a Notch activator) failed to rescue angiogenic defects, whereas guanosine supplementation and MAPK activation effectively alleviated MMF-induced vascular abnormalities, indicating that MMF disrupts zebrafish angiogenesis primarily by impairing IMPDH-dependent guanine nucleotide synthesis and MAPK signaling.

The vasculature, one of the first systems to emerge during vertebrate embryonic development, consists of the heart, blood vessels, and lymphatic vessels. Its main role is to transport oxygen, nutrients, metabolic wastes, and other biomolecules to all cells of the body. The proper formation and connectivity of the vascular network, a continuous process that can meet the changing physiological needs of embryonic development, is an integral part of other organ-forming processes and is essential for embryonic survival [29, 39]. During angiogenesis, neovascularization occurs when mesoderm-derived endothelial progenitor cells, initially present in the lateral plate mesoderm, begin to migrate to the embryonic midline during angiogenesis. When these cells reach the midline, the first circulatory pathway consisting of the dorsal aorta and the main vein begins [10]. These endothelial progenitor cells form blood vessels through two pathways. One is vasculogenesis, which forms blood vessels via de novo synthesis, and the other is angiogenesis, which involves the development of new vessels from existing blood vessels [36]. MMF is teratogenic, and fetuses born to pregnant women with a history of MMF exposure during pregnancy have been shown to have various defects [7]. Angiogenesis is regulated by various factors, and unlike other animal models, zebrafish can survive even in the presence of severe organ defects (such as heart defects) in the form of in vitro diffusion. Most importantly, almost all of the tools that are used to study the cardiovascular system in other model systems can be employed to study zebrafish models [38]. Therefore, the use of zebrafish to assess embryonic angiogenetic toxicity greatly improved the survival rate of the model, enabled the study to proceed smoothly, and because zebrafish has a short growth cycle, the experiment period is greatly shortened.

Angiogenesis is an extremely complex process, and it is mainly regulated by various growth factors and their related receptor tyrosine kinases, some of which play a pro-angiogenic or anti-angiogenic role [23]. VEGF is a very potent pro-angiogenic factor that participates in pathological angiogenesis such as in tumor growth and metastasis, rheumatoid arthritis, myocardial ischemia, and others. VEGFa, the most important and effective stimulator of angiogenesis, is associated with angiogenesis and neoangiogenesis and can cause cell proliferation, inhibit apoptosis, increase vascular permeability, dilate blood vessels, recruit inflammatory cells to the site of injury, and other effects [6, 20]. VEGFc is abundantly expressed in embryonic tissues where lymphatic vessels begin to develop, and it combines with VEGFR3 expressed on lymphatic endothelial cells to promote lymphatic vessel development. VEGFd has similar properties to VEGFc and is involved in lymphangiogenesis but not in angiogenesis [22]. FLT-1 and FLT-4 are receptor tyrosine kinases, also known as VEGFR1, that bind to VEGFa and regulate angiogenesis [25]. Flt4 can bind to VEGFc and participate in the regulation of lymphatic vessels and blood vessels [19]. Meanwhile, the tyrosine kinases kdr and kdrl are high-affinity VEGF receptors, similar to Flt1, which play an important role in angiogenesis and vascular development [5, 11, 12]. The present study results showed that the expression level of VEGF-related genes (VEGFaa, VEGFab, VEGFc, and VEGFd) and tyrosine kinase-related genes (FLT-1, FLT-4, Kdr, and Kdrl) decreased to varying degrees. These results suggest that MMF-induced ISV hyperbranching in zebrafish do not involve VEGF signaling.

The MAPK cascade is a key signaling pathway that regulates many cellular processes, and among the many intracellular signaling pathways, the MAPK pathway is involved in cell proliferation, differentiation, apoptosis, angiogenesis, and tumor metastasis [14]. MAPK is divided into four distinct cascades: extracellular signal-associated kinases (ERK1/2), Jun N-terminal kinases (JNK1/2/3), p38,and ERK5 [8, 27, 34, 40, 43]. The current study showed that MAPK1 (ERK2), MAPK3 (ERK1), MAPK9 (JNK2), MAPK8b, p38a, and p38b, supporting the notion that MMF induces ISV hyperbranching by suppressing MAPK signaling.

IMPDH is a rate-limiting enzyme in the de novo synthesis pathway of guanine nucleotide, which participates in several biological pathways. IMPDH has two isoforms in mammals: impdh1 and impdh2. Mutations in either isoform can induce disease. While mutations in impdh1 are associated with retinal degeneration, those in impdh2 are associated with neurodevelopmental disorders [1, 18]. MMF, as an impdh inhibitor, affects the proliferation of guanosine and deoxyguanosine nucleotides in T and B lymphocytes, thereby inhibiting the production of immunoglobulins [37]. Our rescue experiments showed that exogenous guanosine normalized vascular development and abolished ISV hyperbranching, confirming that MMF induces vascular dysplasia via IMPDH inhibition and GTP insufficiency.

Collectively, our data reveal that MMF exposure induces region-specific vascular abnormalities 

in zebrafish, manifesting as excessive intersegmental vascular branching and reduced cerebrovascular growth. To explain this intriguing divergent phenotype, we propose mechanistic hypotheses supported by published studies: differential basal expression of IMPDH isoforms between trunk and cerebral endothelial cells may cause heterogeneous sensitivity to GTP depletion [2, 3]; distinct MAPK isoform profiles across vascular beds may lead to opposite angiogenic outcomes following pathway inhibition [4, 21, 41]; tissue-specific VEGF signaling thresholds may contribute to the discrepant vascular responses [26]. The underlying mechanism remains a limitation of this study and warrants further in-depth investigation.

## Conclusion

The current study results showed that MMF causes vascular developemnt disturbances by IMPDH and MAPK signaling inhibition. This study provides a theoretical basis for the clinical use of MMF.

## Data Availability

All of the data presented in this study could be obtained from corresponding author.
